# Type II Interleukin-4 Receptor Activation in Basal Breast Cancer Cells Promotes Tumor Progression via Metabolic and Epigenetic Modulation

**DOI:** 10.3390/ijms25094647

**Published:** 2024-04-24

**Authors:** Demond Williams, Ebony Hargrove-Wiley, Wendy Bindeman, Daniel Valent, Adam X. Miranda, Jacob Beckstead, Barbara Fingleton

**Affiliations:** 1 Program in Cancer Biology, Department of Pharmacology, Vanderbilt University, Nashville, TN 37232, USA; demond.williams@vanderbilt.edu (D.W.); ebony.l.hargrove-wiley@vanderbilt.edu (E.H.-W.); wendy.e.bindeman@vanderbilt.edu (W.B.); daniel.valent@vanderbilt.edu (D.V.); adam.x.miranda@vanderbilt.edu (A.X.M.); 2 Department of Pharmacology, Vanderbilt University, Nashville, TN 37232, USA; jacob.a.beckstead@vanderbilt.edu

**Keywords:** interleukin-4, interleukin-13, glucose, histone acetylation

## Abstract

Interleukin-4 (IL4) is a Th2 cytokine that can signal through two different receptors, one of which—the type II receptor—is overexpressed by various cancer cells. Previously, we have shown that type II IL4 receptor signaling increases proliferation and metastasis in mouse models of breast cancer, as well as increasing glucose and glutamine metabolism. Here, we expand on those findings to determine mechanistically how IL4 signaling links glucose metabolism and histone acetylation to drive proliferation in the context of triple-negative breast cancer (TNBC). We used a combination of cellular, biochemical, and genomics approaches to interrogate TNBC cell lines, which represent a cancer type where high expression of the type II IL4 receptor is linked to reduced survival. Our results indicate that type II IL4 receptor activation leads to increased glucose uptake, Akt and ACLY activation, and histone acetylation in TNBC cell lines. Inhibition of glucose uptake through the deletion of Glut1 ablates IL4-induced proliferation. Additionally, pharmacological inhibition of histone acetyltransferase P300 attenuates IL4-mediated gene expression and proliferation in vitro. Our work elucidates a role for type II IL4 receptor signaling in promoting TNBC progression, and highlights type II IL4 signaling, as well as histone acetylation, as possible targets for therapy.

## 1. Introduction

Breast cancer is the most frequently diagnosed cancer amongst women, and the second leading cause of cancer death [1]. A particular concern in the treatment of breast cancer is the elevated mortality rate of metastatic disease [2]. Triple-negative breast cancer (TNBC) is a subset of breast cancer that lacks expression of estrogen receptor, progesterone receptor, and human epidermal growth factor receptor 2. Patients diagnosed with TNBC have a worse prognosis than those with other subtypes of breast cancer, with a 5 year survival rate of 12% for those diagnosed with metastatic disease. This compares with a 5 year survival rate of 32% for those diagnosed with metastatic breast cancer of other subtypes [1]. Additionally, TNBC shows increased rates of relapse and metastasis [3,4,5]. This is likely owing to a lack of targeted therapies for triple-negative breast cancer, in contrast to the various options available for other subtypes. While some targeted therapies, particularly immunotherapies, including immune checkpoint blockade and adoptive T cell transfer, have found success in early trials for the treatment of TNBC, there remains a distinct difference in outcomes [6]. Therefore, the identification of avenues for efficacious targeted therapy in the TNBC setting is critical to improve patient outcomes.

Cytokines such as interleukins are associated with breast cancer progression and metastasis [7]. Several of these or their receptors, such as the IL6 receptor, have been investigated as therapeutic targets [8]. Here, we focus on the receptor for the Th2 cytokines IL4 and IL13. Levels of the type II IL4 receptor, the version predominantly expressed by non-hematopoietic cells, are upregulated in breast cancer [9]. We have previously shown that IL4 receptor signaling contributes to metastasis in mouse models of breast cancer [10]. Additionally, IL4 signaling has been demonstrated to contribute to the progression of prostate [11], colorectal [12], and hepatocellular carcinoma [13]. Both IL4 and IL13 can act as ligands for the type II IL4 receptor and lead to the activation of STAT6, PI3K/Akt and MAPK pathways with downstream effects on multiple cellular processes [14].

To understand how IL4 signaling supports the metastatic progression of breast cancer, it is important to understand the downstream effects of IL4R activation in this context. In previous work, we identified metabolic changes, including increased glucose uptake, in breast cancer cells stimulated with IL4 [15]. A similar phenotype has also been described in pancreatic cancer [16]. Additionally, IL4 signaling in macrophages was linked to epigenetic alterations responsible for the activation and function of macrophages [17]. Here, we assess how these previously identified metabolic and epigenetic effects of IL4 signaling are active in epithelial TNBC cells. We explore how these effects are linked and can result in enhanced proliferative and survival signaling that could promote breast cancer metastasis.

## 2. Results

### 2.1. Type II IL4R Is Associated with Reduced Survival of Basal Breast Cancer Patients

**Figure 1:**
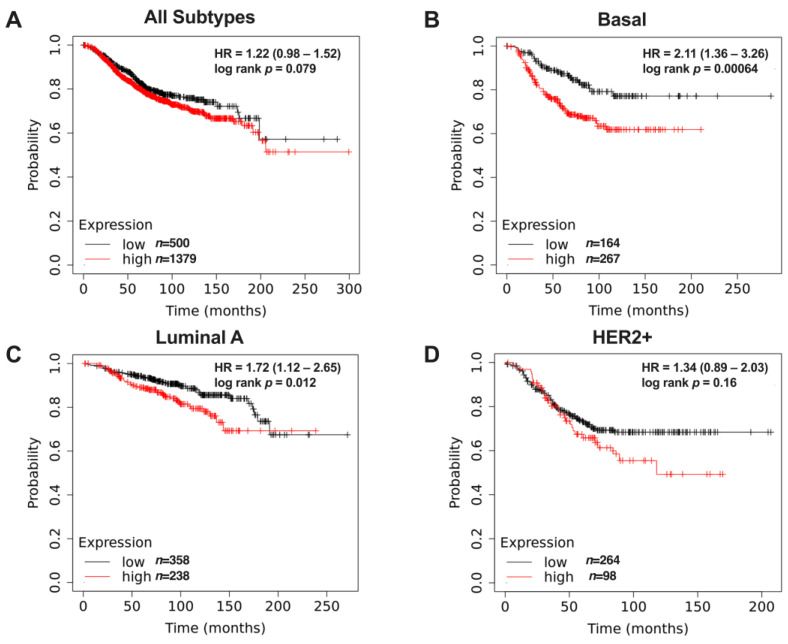
IL13RA1 expression and overall survival from KM plotter (www.kmplot.com, accessed on 16 April 2024) using publicly available data, as described [17]. (**A**) In patients with all subtypes (*n* = 1879), overall survival is not statistically different between patients whose tumors have high versus low IL13RA1 expression. (**B**) High IL13RA1 expression in tumors from patients with the basal subtype of breast cancer (*n* = 431) have significantly reduced survival. (**C**) IL13RA1 expression in tumors from patients with the Luminal A subtype (*n* = 596) moderately impacts survival. (**D**) In HER2+ patients (*n* = 362), IL13RA1 expression does not correlate with survival.

In order to establish the relevance of type II IL4 signaling in breast cancer, we investigated the correlation of the different receptor subunits’ expression to survival in breast cancer patients using publicly available datasets [18]. Of the two components of the type II IL4 receptor (IL4R and IL13RA1), IL13RA1 is unique to this receptor, while IL4R is also found extensively on leukocytes as part of the type I IL4 receptor complex [19]. Therefore, we used the expression of IL13RA1 as a proxy for type II IL4R levels. IL13RA1 expression was not correlated with survival across all breast cancer subtypes (Figure 1A); however, there was a significant difference in survival observed particularly in the basal subtype (Figure 1B) and to a lesser extent in the luminal A subtype (Figure 1C). The HER2+ subtype showed no significant correlation between IL13RA1 expression and decreased survival (Figure 1D). Conversely, high expression of type I IL4 receptor subunits (IL4R and IL2RG) showed a slight association with better survival in the same dataset (Figure S1), which may reflect a link between immune cell infiltration and the outcome. Taken together, the data suggest that the type II IL4 receptor is a poor prognostic indicator, especially in the context of TNBC or basal breast cancer.

### 2.2. IL4 Increases Glucose Uptake and Metabolism

**Figure 2:**
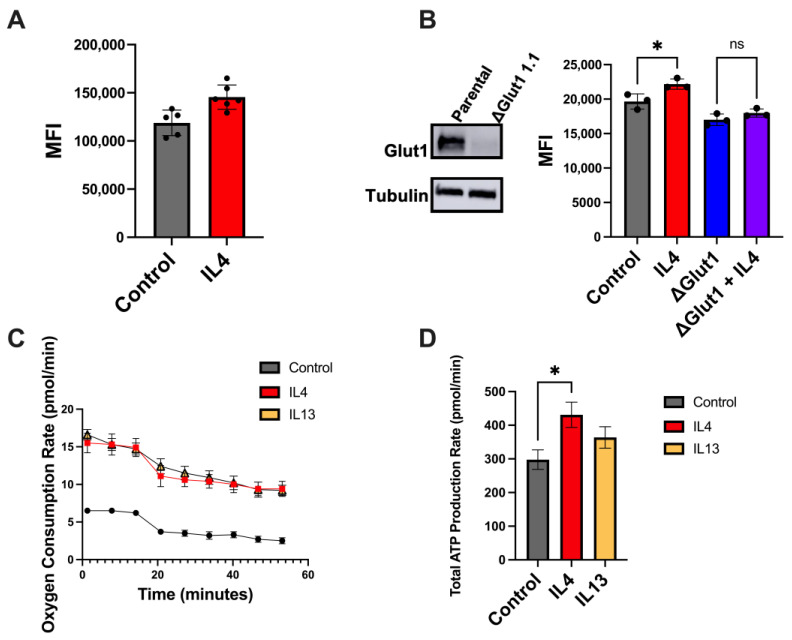
IL4 increases glucose uptake and metabolism in TNBC cell lines. (**A**) Treatment with 10 ng of IL4 increases glucose uptake in 4T1 murine mammary cancer cells, as assessed by the NBDG assay. (**B**) The Glut1 protein is undetectable in ΔGlut1 MDA-MB-231 cells generated using CRISPR/Cas9, and treatment with 20 ng of IL4 shows that an IL4-induced increase in glucose uptake is dependent on GLUT1 expression. (**C**) Pre-treatment (24 h) with 20 ng of IL4 or IL13 results in increased oxygen consumption rates in MDA-MB-231 cells, as measured by the Seahorse ATP rate assay. (**D**) Pre-treatment (24 h) with 20 ng of IL4 or IL13 results in increased total ATP production in MDA-MB-231 cells, as measured by the Seahorse ATP rate assay. Seahorse readings were normalized to the cell number. Representative images from experiments that were repeated at least 3 times are shown. * *p* < 0.05, ns = non-significant.

Type II IL4 receptor signaling has been previously associated with elevated glycolysis in breast cancer [15] and other cancer types [16]. These IL4-induced changes in metabolism have been associated with increased metastasis. To assess these metabolic effects on triple-negative breast cancer in vitro, we treated two cell lines capable of spontaneous metastasis, human (MDA-MB-231) and murine (4T1) cell lines, with IL4 and quantified glucose uptake using the fluorescent glucose analog 2-NBDG. Using this assay, we found that IL4 increased glucose uptake in both cell lines (Figure 2A,B). Additionally, we have previously shown that IL4 increases expression of the glucose transporter GLUT1 in breast cancer cell lines [15]. Using RNA-mediated knockdown of GLUT1, we found that IL4-induced glucose uptake was dependent on GLUT1 expression (Figure 2B).

To assess the potential impact of type II IL4 signaling on energy production, we used a Seahorse ATP rate assay to measure the glucose consumption rate of MDA-MB-231 cells pre-treated with IL4 for 24 h. Interestingly, we found that both of the ligands for the type II Il4 receptor—IL4 and IL13—consistently increased the oxygen consumption rate (OCR) of these cells compared to untreated controls (Figure 2C). However, changes in the extracellular acidification rate were not consistent across replicate assays. Additionally, total ATP production rates were only marginally increased in cells treated with these cytokines (Figure 2D). Similar results were seen with the BT549 cells, another human triple-negative breast cancer cell line (Figure S2). Overall, the data suggest that IL4 increases glucose uptake, but only leads to small increases in lactate production or ATP production in the time frames examined. For this reason, we next considered other possible fates of glucose.

### 2.3. The pAKT/pACLY Signaling Axis Is Activated by IL4 Signaling

Glucose can be used to produce acetyl-CoA, which, among other roles, is a substrate for histone acetylation [20]. Histone acetylation is well established to be dependent on the abundance of acetyl-CoA [21]. IL4 can regulate Akt activation, which in turn results in serine 455 phosphorylation and increased activity of ATP-citrate-lyase (ACLY), an enzyme that produces acetyl-CoA from the TCA cycle intermediate citrate [17]. In macrophages, IL4 increases glucose consumption and ACLY activity necessary for the regulation of gene expression and polarization [22].

**Figure 3:**
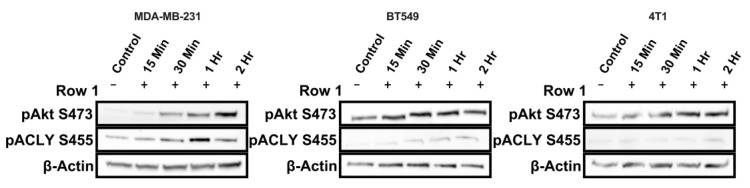
IL4 treatment induces the phosphorylation of Akt and ATP citrate lyase (ACLY). Treatment with 20 ng of species-specific IL4 results in increased phosphorylation of AKT at serine 473 and ACLY at serine 455 over time in human MDA-MB-231, human BT549, and murine 4T1 cells. Representative images from at least 2 repeats per cell line are shown.

Since we observed increased glucose uptake with IL4 treatment, we investigated whether the pAKT/pACLY signaling axis was also affected by IL4 signaling in the context of breast cancer. An important precursor to the phosphorylation of ACLY is the phosphorylation of AKT at serine 473. We found that IL4 induced the phosphorylation of AKT and ACLY in both human and murine mammary cancer cell lines (Figure 3). Together, this suggests that type II receptor signaling may affect the utilization of glucose to support IL4-induced histone acetylation and gene expression in triple-negative breast cancer.

### 2.4. Histone Acetylation Leading to Gene Expression Changes Is Altered by IL4 Signaling

**Figure 4:**
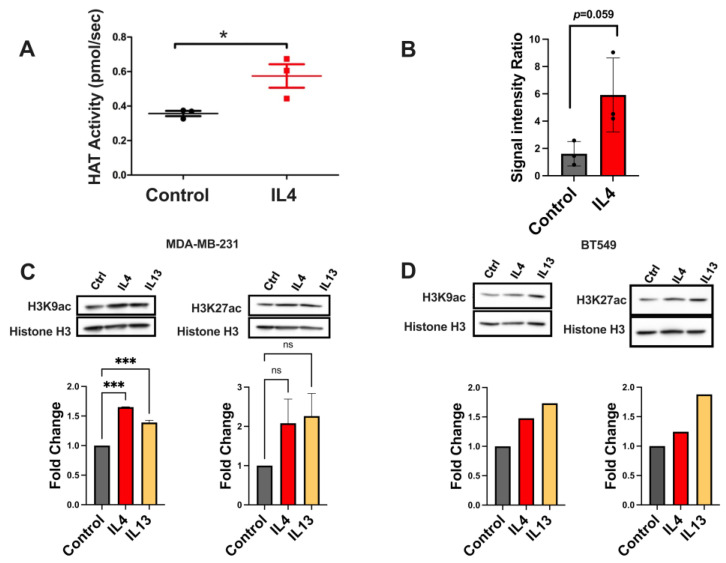
Stimulation of type II IL4 receptor increases histone acetylation in triple-negative breast cancer. (**A**) HAT activity assessed by a fluorometric assay in nuclear lysates from MDA-MB-231 cells shows increased HAT activity with IL4 treatment. (**B**) Acetyl-lysine pull down was performed on total lysates from MDA-MB-231 cells. Western blots for total histone H3 were performed on enriched lysates showing an increased presence of histone H3 after IL4 treatment. Western blots for H3K27ac and H3K9ac in MDA-MB-231 (**C**) and BT549 (**D**) cells showed moderate increases in global acetylation at these marks of interest. Quantification of blots is shown below (n = 2 for MDA-MB-231 cells, n = 1 for BT549 cells). * *p* < 0.05; *** *p* < 0.001, ns = non-significant.

After confirming the activation of ACLY, we next investigated whether IL4 impacts histone acetylation in TNBC. We first examined whether there were alterations in overall histone acetyltransferase (HAT) activity in nuclear lysates from TNBC cell lines with or without IL4 exposure. We found that IL4 increased HAT activity, as demonstrated by a fluorescent HAT activity assay (Figure 4A).

Next, we were interested in whether IL4 induces global changes in histone acetylation. We first enriched acetyl-lysine proteins using immunoprecipitation, and then assessed their association with histone H3 by immunoblotting. Our results indicated that lysates from IL4-treated cells were enriched in acetyl-lysine-positive histone H3 (Figure 4B). We then investigated specific acetylation marks that have been associated with IL4-mediated gene regulation [23]. Histones that were isolated from IL4-stimulated cell lines showed small increases in global acetylation of H3K9 and H3K27 acetylation compared with controls (Figure 4C).

Due to the moderate increases in global histone acetylation, we focused on the role of IL4 in the modulation of acetylation at the gene level. IL4-mediated gene expression is dependent on the association of IL4 transcription factors such as STAT6 with cofactors such as the histone acetyltransferase p300 [24]. Thus, large changes in acetylation across the genome are less likely than more precise regulation of STAT6 target genes. To investigate this, we performed CUT&RUN (cleavage under targets and release using nuclease) sequencing of the MDA-MB-231 cell line to identify STAT6 and H3K9ac target genes that are regulated by IL4. In tandem, we investigated which genes are altered in response to IL4 treatment in MDA-MB-231 cells using RNASeq. Due to our interest in histone acetylation, which is a permissive epigenetic marker, our focus was largely on genes that showed increased expression with IL4 stimulation.

**Figure 5:**
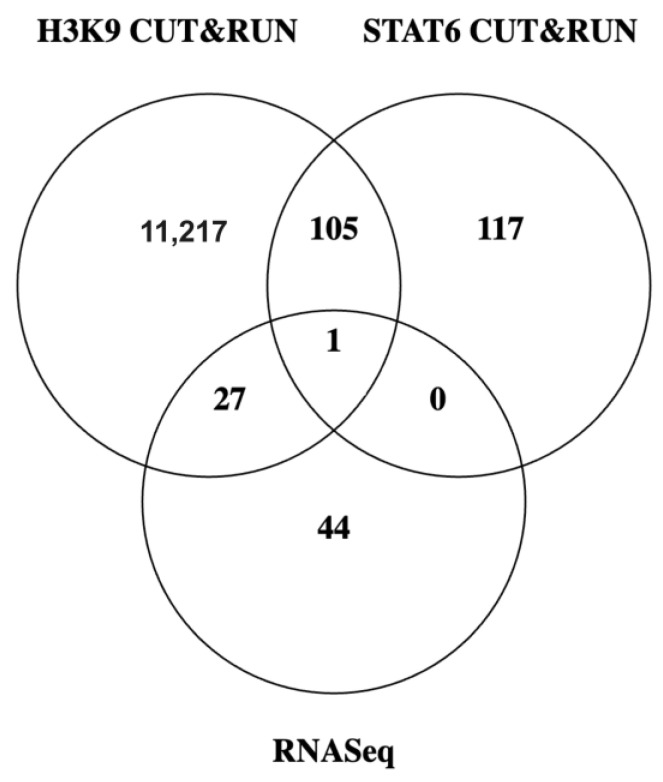
Overlap in genes identified by sequencing experiments. Gene lists from RNASeq experiments that showed increased expression, and H3K9ac and STAT6 target genes found using CUT&RUN were intersected in order to identify IL4-induced genes that are regulated by histone acetylation.

**Figure 6:**
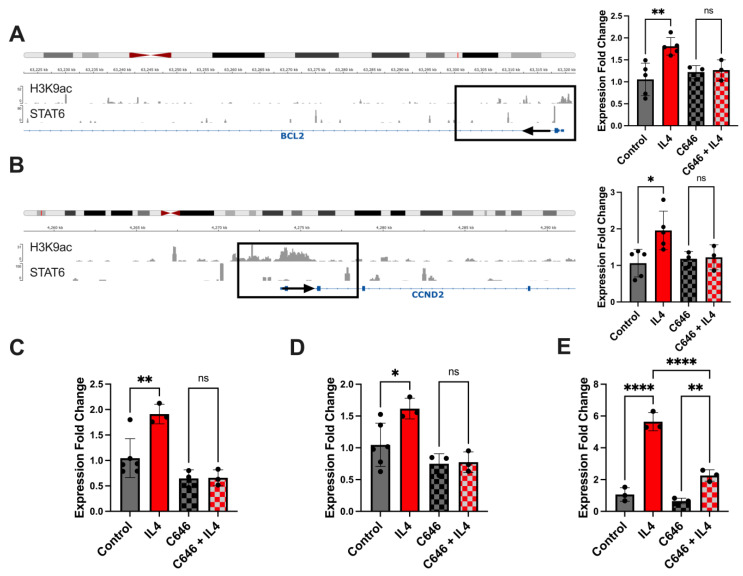
IL4 induces the expression of target genes in a histone acetyltransferase-dependent manner. (**A**,**B**) Integrative genome viewer views of signals from H3K9ac and STAT6 CUT&RUN experiments overlapping with BCL2 and CCND2 promoter regions (boxed). Black arrows indicate direction of gene from start site. Results of the quantitative RT-PCR analysis of the expression of the transcripts are shown to the right. (**C**–**E**) Quantitative RT-PCR for TNC, EP300, and ST8SIA1, respectively, in MDA-MB-231 cells. For all qRT-PCR experiments, cells were treated with 20 ng of recombinant IL4 with or without 20 μM C646 for 24 h before RNA collection. Results from three biological replicates are shown. * *p* < 0.05; ** *p* < 0.01; **** *p* < 0.0001; ns = non-significant.

From the intersection of the datasets generated by these experiments (Figure 5), we developed a list of candidate genes whose expression was mediated by IL4 and showed a dependence on histone acetylation. Within that gene set, we focused on those that are known oncogenes or have been associated with metastatic progression in other ways in the literature. Five genes satisfied these criteria and were subject to further validation: BCL2, CCND2, TNC, ST8SIA1, and EP300. Figure 6A,B shows examples of overlap in location of the H3K9 acetylation and STAT6 binding signals within two of these genes. We examined expression of genes of interest in cells treated with or without IL4 in the presence or absence of C646, a small-molecule inhibitor of the STAT6-associated HAT p300 [25] (Figure 6A–E). For all transcripts examined, C646 alone did not decrease the expression of these genes below the baseline. However, in cells exposed to IL4, C646 attenuated the IL4-induced expression of CCND2, BCL2, TNC, ST8SIA1, and EP300.

### 2.5. Both p300 and Glucose Uptake Contribute to Type II IL4R-Induced Proliferation and Survival

Several of the genes of interest identified in our sequencing experiments and confirmed by qPCR are known to be associated with cancer progression. CCND2 is a cell cycle protein that has been associated with progression in colorectal cancer [26]. BCL2 is a known target of IL4 signaling [27] and an important regulator of apoptosis that has become a target of interest in several cancers [28]. We have previously shown that IL4 stimulation causes increased proliferation and clonogenicity, which are in vitro surrogates for metastatic phenotypes closely associated with these genes [15]. Due to the impact of C646 on the expression of these genes, we next investigated whether C646 could ablate the IL4-induced phenotypes.

**Figure 7:**
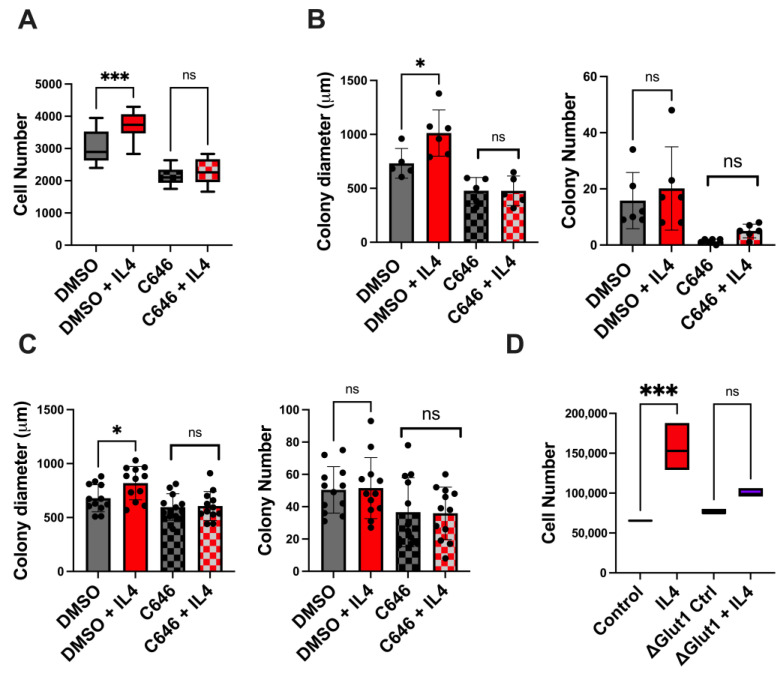
IL4-induced in vitro phenotypes are dependent on p300 activity and glucose uptake. (**A**) Cyquant proliferation assay showing increased numbers of BT549 cells treated with 10 ng of IL4 for 24 h. The inclusion of 10 μM C646 ablates the IL4-induced increase in cells. (**B**) The clonogenic assay of BT549 cells shows an increased colony size in cells treated with 10 ng of IL4, while the inclusion of 10 μM C646 ablates the effect of IL4 on the colony size. Cells were treated every 2 days for a total of 14 days in culture. (**C**) Murine mammary cancer 4T1 cells show an increased colony size after treatment with 10 ng of IL4, which is prevented by the inclusion of 10 μM C646. The colony number was unaffected by IL4. Cells were treated every 2 days for a total of 6 days in culture. (**D**) The Cyquant assay of MDA-MB-231 parental and Glut1 knockout cell lines shows that IL4-mediated proliferation is also dependent on glucose uptake. * *p* < 0.05; *** *p* < 0.001; ns = non-significant.

In regard to cell proliferation, we found that treatment with IL4 increased the cell number, as measured by the Cyquant assay, and C646 ablated the IL4 induced proliferation in BT549 cells (Figure 7A). We also performed clonogenic assays with both BT549 and 4T1 cell lines to assess the impacts of IL4 and C646 on cell proliferation and clonogenicity. MDA-MB-231 cells were not used for these assays as they are highly migratory and do not form cohesive colonies. While IL4 did not impact the number of colonies, the average colony size was significantly increased with IL4 treatment (Figure 7B,C), supporting proliferation as an IL4-induced phenotype. C646 treatment alone did not affect the size of the colonies formed; however, it did ablate the effect of IL4 on the increased colony size.

Using Glut1-deficient MDA-MB-231 cells, we also investigated whether IL4 treatment increased proliferation in a manner dependent on glucose uptake. As shown in Figure 7D, treatment of the parental MDA-MB-231 cells with IL4 increased proliferation, as determined by the Cyquant assay. However, the stimulatory effect of the cytokine was no longer observed in the cells lacking Glut1. Together these data support the idea that type II IL4 receptor signaling leads to the increased survival and proliferation of TNBC cells via a mechanism involving glucose uptake and histone acetylation.

## 3. Discussion

Here, we show that type II IL4 receptor signaling can contribute to metastatic phenotypes, including proliferation and clonogenic survival, in triple-negative breast cancer. Our data indicate that activation of the type II receptor by either of the cognate ligands—IL4 or IL13—leads to increased glucose uptake, which is associated with enhanced proliferation. Additionally, a direct result of receptor activation is the phosphorylation of Akt and ACLY, which has been linked to increased availability of acetyl-CoA, a substrate for histone acetyltransferases. Histone acetyltransferase activity is increased in IL4-treated cells, as are levels of lysine-9 and -27 acetylation on histone 3 albeit only moderately on a global level. Specific IL4 target genes of relevance in breast cancer metastasis were identified by RNASeq and CUT&RUN experiments and show dependence on P300 histone acetyltransferase activity for their IL4-driven expression. Together, these in vitro findings enhance our previous in vivo data [10], which showed significantly attenuated metastasis when the IL4 receptor was ablated in cancer cells. The current data suggest that changes to glucose uptake and histone acetylation are potentially key effectors of IL4 signaling in metastatic breast cancer cells.

Many of the IL4-mediated phenotypes investigated have been observed in various immune cells where IL4 generally signals through the type I IL4 receptor. For example, gene expression associated with M2 polarization of macrophages is dependent on IL4-driven glucose metabolism [29], and blocking glycolysis using 2DG impairs macrophage polarization [30]. In addition to altered gene expression, 2DG treatment also impacts phagocytosis [31]. In B-lymphocytes, IL4 has an anti-apoptotic effect that is also dependent on glycolysis [32], while the activation of T-lymphocytes driven by autocrine IL4 signaling is similarly dependent on glycolysis [33]. Other metabolic pathways modulated by IL4 have also been associated with macrophage function. IL4-driven M2 polarization appears to be dependent on a certain energy threshold, with glutaminolysis and oxidative phosphorylation acting as compensatory metabolic pathways if glycolysis is inhibited [34]. Serine biosynthesis pathways have additionally been implicated, with genetic ablation of PHGDH resulting in attenuated induction of M2 polarization by IL4 in vivo [35]. We have also previously shown using pharmacological inhibitors that glutaminolysis in addition to glycolysis are tied to IL4-induced proliferation in breast cancer [15]. We extend that finding here using CRISPR-mediated deletion of GLUT1, which ablates IL4-induced proliferation in TNBC. Overall, the mechanisms by which IL4 signaling drives the proliferation of breast cancer cells align well with those observed in other cell types. Of note, based on the data shown here, we can expand that model to include signaling through the type II Il4 receptor.

The Akt/ACLY signaling axis has been implicated as an important mediator of IL4-induced polarization of murine macrophages [17]. This signaling links both glycolysis and mitochondrial metabolism to gene expression in M2 polarized macrophages [22]. In human macrophages, while full polarization is not dependent on ACLY, some IL-4-induced gene expression is still reliant on ACLY activity, as indicated by pharmacological inhibition of ACLY [36]. ACLY is a substrate of Akt, and we noted increased Akt activation in the cells treated with either IL4 or IL13. This is in line with data that we published previously showing that IL4-regulated metastatic colonization in mouse models of breast cancer was linked to increased Akt signaling, and IL4-enhanced colony formation in vitro was attenuated by Akt inhibition [10]. We acknowledge that the Akt/ACLY signaling axis may play several other roles in TNBC tumor progression, for example, in DNA repair [37]; however, here, we chose to focus on histone acetylation as a potentially relevant downstream effect.

Previous evidence suggests that ACLY phosphorylation and activity regulate gene expression via histone acetylation in macrophages [17] and osteoclasts [38]. Moreover, IL4-driven histone acetylation is an important regulator of gene expression in B-lymphocytes [23] and macrophages [39,40], and the histone acetyltransferase P300/CBP is well recognized as an important mediator of IL4-stimulated gene regulation [41,42]. In our hands, IL4 treatment induced relatively small increases in total histone acetylation. However, using more targeted methods, we could identify IL4 target genes where histone acetylation was active. Other epigenetic modifications are known to be modulated by IL4 in immune cells, such as histone deacetylation [43,44], histone demethylation [45,46,47], and DNA demethylation [48]. In this study, we did not examine the contributions of these modifications; however, it is likely that they play a role. A future goal is to systematically characterize epigenetic changes that occur downstream of IL4/IL13 signaling in cancer cells.

C646 has been previously investigated as a potential drug for pancreatic cancer [49], hepatocellular carcinoma [50], and prostate cancer [51]. Here, we show that inhibition of the histone acetyltransferase p300 by C646 blocks IL4-driven gene expression in TNBC cell lines. Specifically, we have identified CCND2, BCL2, TNC, ST8SIA1, and EP300 as genes upregulated by IL4 in a p300-dependent manner. Expression of CCND2 has been shown to be correlated with persistence of colorectal cancer [26]. BCL-2 family proteins have been identified as targets for cancer therapy [28], and their expression has been shown to be induced by IL4 in lymphocytes [27]. The expression of both genes is in line with the IL4-induced proliferation and survival phenotypes observed in this study. Tenascin C (TNC) expression has been associated with multiple cancer processes, including enhanced proliferation, migration [52], and immunosuppression [53]. ST8SIA has previously been shown to play a role in chemoresistance in triple-negative breast cancer by modulating Wnt/β-catenin signaling [54]. EP300 has been associated with the metastatic capacity of triple-negative breast cancer [55]. In addition to effects on gene expression, C646 treatment attenuates metastatic phenotypes in IL4-treated TNBC cell lines in vitro.

The work presented here was conducted in vitro, and there is a need for further investigations using in vivo models to provide a better understanding of the varied roles of IL4 signaling and its contribution to cancer progression. In the context of immune cells, IL4 signaling has been characterized as largely immunosuppressive and pro-tumorigenic, mediated by the previously discussed role it plays in polarizing tumor-associated macrophages to the M2 phenotype [56]. However, there is some evidence in the literature to suggest that IL4 signaling acting through CD8 lymphocytes expressing the type I IL4 receptor can boost anti-tumor immunity in the context of immune checkpoint blockade [57]. Here, we demonstrate that IL4 signaling acting on breast cancer cells through the type II receptor is mitogenic and supports the expression of pro-survival genes. These context-dependent effects of IL4 signaling highlight the importance of specificity in targeting the IL4 signaling axis in vivo. Experiments using specific inhibitors of type II IL4 receptor [58] vs. type I IL4 receptor in immune-competent mouse models of breast cancer would be necessary to elucidate the how the two receptors may contribute to tumor progression. In addition, it would be necessary to characterize immune cells in the TME and how they respond to modulation of the IL4 receptors, as well as the signaling consequences of targeting IL4 in cancer cells themselves.

As shown by the drastic decrease in five-year survival rates seen in patients diagnosed with breast cancer at later stages [2], metastatic disease remains a particularly significant clinical challenge. In addition, triple-negative breast cancer is often aggressive, and there exist relatively few targeted therapies for this subtype. Therefore, the identification of additional vulnerabilities of these tumors could greatly benefit patient care. In the context of cancer, IL4 signaling has largely been studied for its effects on immune cells that drive cancer progression. IL4-driven polarization of macrophages has been associated with immunosuppression in breast cancer [56]. In addition, macrophages polarized by IL4 signaling secrete factors that promote the invasion and migration of breast cancer [59]. Repurposing of dupilumab, an IL4R-targeted antibody, has shown promising synergy with immune checkpoint inhibitors in the treatment of non-small cell lung cancer [60]. The data presented here support a direct effect of IL4 signaling on triple-negative breast cancer mediated through the type II IL4 receptor. This is in addition to the better studied role of IL4 signaling in immune cells, driving immunosuppression and tumor progression indirectly.

## 4. Materials and Methods

**Reagents:** Primary antibodies used in these studies include those targeting pAkt S473 (Cell Signaling Technology, Danvers, MA, USA, #4060S), total Akt (Cell Signaling Technology #4691S), pACLY S455 (Cell Signaling Technology #4331S), total ACLY (Cell Signaling Technology #13390S), B-Actin (Cell Signaling Technology #5125S), STAT6 (Santa Cruz Biotechnology, Dallas, TX, USA, sc-374021), H3K9ac (Active Motif, Carlsbad, CA, USA, #91103), H3K27ac (Active Motif #39133), and Histone H3 (Active Motif #39763). Control and secondary antibodies were anti-rabbit IgG, HRP-linked antibody (Cell Signaling Technology #7074S), and CUTANA IgG negative control antibody (Epicypher, Cambridgeshire, UK, Cat# 13-0042). Other reagents used were CUTANA pAG-MNase for CHIC/CUT&RUN workflows (Epicypher Cat# 15-1016), C646 (Selleck, Houston, TX, USA, Cat#S7152), NBDG (Caymen Chemical, Ann Arbor, MI, USA, #11046), human IL4 (Peprotech, Rocky Hill, NJ, USA, #200-04), human IL13 (Peprotech #200-13), human IL4 (BD Biosciences, Franklin Lakes, NJ, USA, #554605), mouse IL4 (Peprotech #214-14), Mouse IL13 (Peprotech #210-13), mouse IL4 (BD Biosciences #550067), and mouse IL13 (BD Biosciences #554599).

**Cell culture:** MDA-MB-231, BT549, and 4T1 cell lines were purchased from the American Type Cell Culture Collection (ATCC, Gaithersberg, MD, USA). The cells were maintained in Dulbecco’s Modified Eagle’s Medium (DMEM; Corning, New York, NY, USA) containing 10% fetal bovine serum (FBS; Atlanta Biologicals, Flowery Branch, GA, USA) and supplemented with gentamicin (Corning) at 37 °C and 5% CO_2_. Cell lines were used below passage 25.

**Generation of GLUT1-deleted cells:** MDA-MB-231 were transfected with ribonucleoprotein complexes formed between a mix of sgRNAs targeted to GLUT1 (Synthego, Redwood City, CA, USA) and the Cas9 protein (Synthego) using Lipofectamine CRISPRMAX reagent (Invitrogen, Waltham, MA, USA) according to the Synthego standard protocol. At 3 days post-transfection, cells were cloned out by limiting dilution. After individual clones had sufficiently grown, samples were processed for GLUT1 detection by western blotting. Clones with confirmed deletion were used for subsequent experiments.

**Preparation of cell lysates and quantification**: Cells were washed in PBS and then scraped into RIPA buffer on ice, and the lysate was transferred to Eppendorf tubes. Samples were spun at 10,000× *g* at 4 °C. Lysates were then transferred to new tubes and kept at −20 °C until use. The total protein concentration of lysates was determined using the BCA assay (Thermo Fisher, Waltham, MA, USA).

**Histone isolation:** Histones were extracted using an acid extraction method [61]. After nuclear fractionation, the nuclear pellet was suspended in 0.4 N H_2_SO_4_ overnight. The nuclear pellet in sulfuric acid was then centrifuged at 16,000× *g*, and histones were precipitated in trichloroacetic acid.

**Western blotting:** Cells were treated with 20 ng of recombinant IL4 or recombinant IL13 prior to total lysate isolation. Cell lysates were run on SDS poly-acrylamide gels and subsequently transferred to nitrocellulose membranes that were then blocked in 5% milk in TBS-T for 1 h at room temperature. The membranes were incubated with the appropriate primary antibody in 5% milk in TBS-T at 4 °C overnight with shaking. This was followed by an incubation with HRP-linked secondary antibodies for 1 h at room temperature with shaking. Signals were detected using Clarity Western ECL Substrate (Biorad, Hercules, CA, USA) with an Amersham Imager 600 instrument (GE Healthcare Life Sciences, Piscataway, NJ, USA). Blots were stripped with Oneminute western blot stripping buffer (GM Biosciences, Frederick, MD, USA) according to the manufacturer’s recommendations and then reprobed with loading control antibodies (anti-β actin or anti-total histone H3, as appropriate).

**Glucose uptake assays:** The NBDG uptake assay was performed as described previously [15].

**Extracellular flux/ATP rate assays**: MDA-MB-231 cells were plated at a density of 10,000 cells per well on XF Cell Culture Microplates (Agilent, Santa Clara, CA, USA) at 37 °C and 5% CO_2_. Cells were treated with 20 ng of human IL4 or 20 ng human IL13 for 24 h in DMEM. At 1 h prior to the assay, the medium was replaced with Agilent Seahorse XF DMEM pH 7.4. The plate was then placed in a CO_2_-free incubator for 1 h at 37 °C. Experiments were run using a Seahorse XFe96 Analyzer (Agilent). An ATP Rate assay kit (Agilent) was used following the manufacturer’s protocol. Cells were stained with Hoechst solution, and ATP rate assay data were normalized to cell number counted using fluorescence.

**HAT activity assay**: MDA-MB-231 cell lines were treated with 10 ng of recombinant human IL4 (BD Biosciences) for 24 h. After treatment, nuclear extracts were collected using NE-PER Nuclear and Cytoplasmic Extraction Reagents (Thermo Scientific). HAT activity was measured using the Histone Acetyltransferase Activity Assay Kit (Abcam, Cambridge, MA, USA) following the manufacturer’s protocol. The protein concentration of nuclear extracts was measured using the BCA assay. HAT activity was normalized to the protein concentration of each sample.

**Acetyl-Lysine immunoprecipitation**: Cells were treated with 10 ng of recombinant human IL4 for 24 h. After being washed with PBS, cells were immunoprecipitated with the Signal Seeker Acetyl-Lysine Detection Kit (Cytoskeleton, Denver, CO, USA, #BK163) following the manufacturer’s instructions.

**CUT& RUN:** MDA-MB-231 cells were plated at a density of 500,000 cells per plate in 60 mm dishes and treated for 48 h with 20 ng of recombinant human IL4 in DMEM. CUT&RUN was performed following the CUTANA (Epicypher) protocol using anti-STAT6, and anti-H3K9ac, and CUTANA negative control antibodies. DNA fragments were generated into libraries using the NEB Next Ultra II DNA library Preparation kit. Sequencing was performed via NovaSeq6000 (PE150) with an average of 10–15 M reads/sample. Library preparation and sequencing were performed by the Vanderbilt University Medical Center VANTAGE core. Raw data were provided as FASTQ files. Trimmomatic was used to remove adapters and low-quality bases from reads [62]. Trimmed reads were then aligned to GRCg38—hg38 genome using bowtie 2 [63] with the following parameters: --local, --very-sensitive, --no-mixed, --no-discordant, --dovetail. Aligned SAM files were converted to sorted BAM files using the SAMtools [64] sort function. PICARD was used to mark duplicate reads in sorted BAM files (https://broadinstitute.github.io/picard, accessed on 15 January 2024). Duplicated and low-quality reads, based on the bowtie2 score, were removed using SAMtools view with the parameters -f 2, -F 1024, and -q 30. Peaks were called using MACS2 [65] over individual replicates using IgG samples as controls. Peak lists were labeled with closest gene name using ChIPpeakAnno [66]. BAM files for biological replicates were merged using the SAMtools merge function. These merged BAM files were converted into BedGraph files normalized to show 1× genome coverage using the Deeptools [67] bamcoverage function. These BedGraph files were used for visualization of the genes of interest in integrative genome viewer (IGV) [68].

**RNA extraction and qRT-PCR:** Cells were treated in serum-free medium. Following treatment, cells were washed with PBS and lysed in TRIzol reagent (Invitrogen). RNA was collected using Quick-RNA Miniprep Kit (Zymo Research, Irvine, CA, USA). cDNA was made using a High-Capacity cDNA Reverse Transcription Kit (Thermo Fisher). qPCR was performed using Power SYBR Green PCR Master Mix (Thermo Fisher) following the manufacturer’s instructions. qPCR was run on a QuantStudio 3 instrument (ThermoFisher). Quantification was performed using the delta delta cT method, using expression of 18S as the reference gene for the normalization of samples. Primer sequences are listed in Supplemental Table S1, except for those for TNC, which were purchased from Realtimeprimers.com.

**RNASeq:** MDAMB231 cells were treated in 10% serum-containing DMEM with 10 ng of human IL4 for 24 or 48 h, and RNA was collected as above. cDNA library preparation was performed using the NEBNext Poly(A) selection library kit. Library quality control was performed using Qubit and bioanalyzer. Sequencing was then performed on the Illumina NovaSeq 6000 platform for paired-end reads of 150 bp, for an average of 50 M reads per sample. The analysis was performed through Illumina’s Dragen RNASeq pipeline. Library preparation, quality control, sequencing, and analysis were performed by VANTAGE.

**Colony-forming assays:** The 4T1 or BT549 cells were plated at 200 cells per well in 12-well plates in 1 mL of medium containing DMSO or 10 μm C646 with or without 20 ng/mL of species-specific IL4. The medium was aspirated and replaced with fresh medium containing the same additives every 2 days. At day 6–7 for 4T1 cells or day 14 for BT549 cells, the plates were aspirated, rinsed with PBS and stained by covering the wells with 1 mL of 0.5% crystal violet containing 6% glutaraldehyde for 30 min at room temperature. The stain was removed, and plates washed by repeated submersion in distilled water before drying overnight. Colony imaging and quantification were performed on the GelCount System (Oxford Optronix, Adderbury, UK) in the Digital Histology Shared Resource at Vanderbilt University Medical Center.

**Proliferation assays**: MDA-MB-231 and BT549 cells were seeded at 5000–10,000 cells per well onto 96 well plates and treated for 24 h with 20 ng of IL4 and/or other drugs, as noted in the figure legends. The cell number was then measured using a Cyquant Direct Cell Proliferation Assay (Thermo Fisher) following the manufacturer’s instructions.

**Prognostic analysis of IL4 receptor subunits in breast cancer:** We accessed the KM plotter web-based tool [18] (www.kmplot.com, accessed on 16 April 2024) to assess the prognostic potential of IL4 receptor subunits and how their expression correlated to overall survival. The publicly available datasets used for this analysis are described in the paper describing the algorithm [17]. All available patient samples were used with no selection for treatment. The probe set used was IL13RA1 201888_s_at, with ‘overall survival’ and auto-select cutoff checked. Selections of the different subtypes were made using the PAM50 designations.

**Statistical analysis**: Analysis was performed using GraphPad Prism version 10.2.0. Student’s t test was used for comparisons between two conditions and one way ANOVA for experiments with 3 or more conditions. *p* < 0.05 was considered statistically significant.

## Data Availability

Datasets from CUT&RUN and RNAseq experiments have been deposited in the Gene Expression Omnibus (GEO) publicly available genomics data repository. Specific accession numbers are in process and will be added. All other raw data are available upon request from the authors.

## Supplementary Materials

The following supporting information can be downloaded at https://www.mdpi.com/article/10.3390/ijms25094647/s1.
