# Supplementary material for: Type II Interleukin-4 Receptor Activation in Basal Breast Cancer Cells Promotes Tumor Progression via Metabolic and Epigenetic Modulation
Source: Int J Mol Sci. 2024 Apr 24;25(9):4647. doi: 10.3390/ijms25094647 (PMC11083536; doi:10.3390/ijms25094647)
Supplement: Supplementary file 1 [file ijms-25-04647-s001.zip › ijms-2939531-supplementary.pdf]

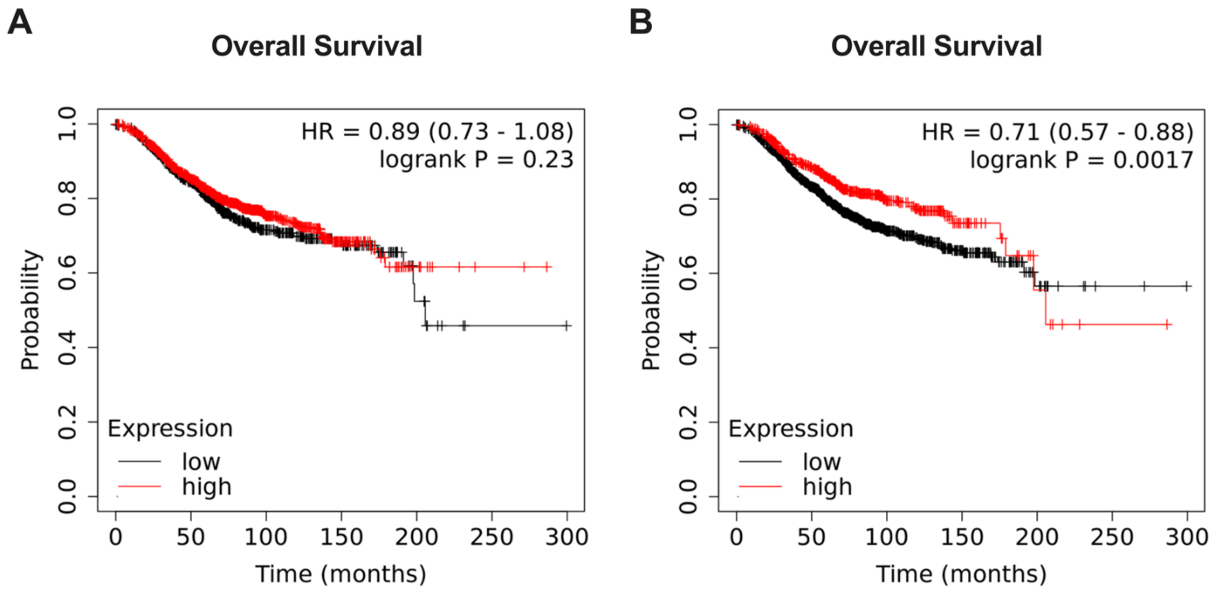

Figure S1. Type I IL4 receptor subunits do not correlate with decreased survival in breast cancer. High expression of neither IL4R (A) nor IL2RG (B) correlates with reduced survival in breast cancer patients that are not stratified by subtype (n=1879). Figure created with KMPl otter.

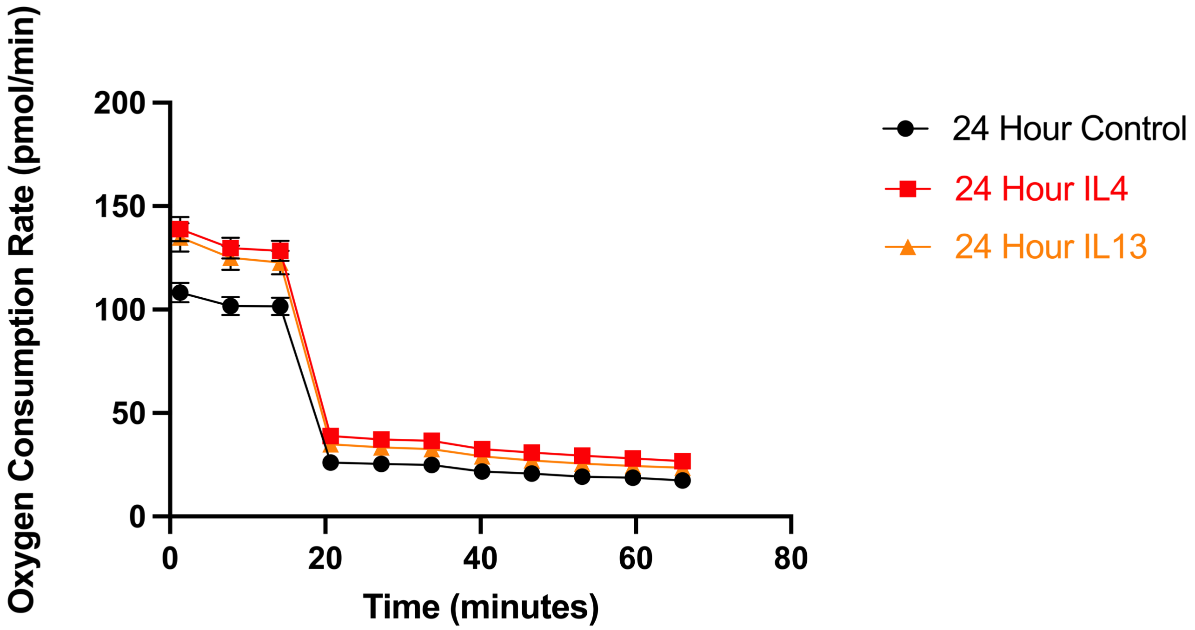

Figure S2. Type II IL4 receptor stimulation increased oxygen consumption rate in BT549 c ells, as assessed by Seahorse glycolytic rate assay.

Supplemental Table S1: Primer sequences

Gene Target	Forward Primer (5' - 3')	Reverse Primer (5' - 3')
18S	CCAGGTCCTAGCAATGGTC	AAGTGACGCAGCCCTCTATG
BCL2	GGTGGGGTCATGTGTGTGG	CGGTTCAGGTACTCAGTCATCC
CCND2	ACCTTCCGCAGTGCTCCTA	CCCAGCCAAGAAACGGTCC
EP300	AGCCAAGCGGCCTAAACTC	TCACCACCATTGGTTAGTCCC
ST8SIA1	GGAAATGGTGGGATTCTGAAG	TGACAAAGGAGGGAGATTGC
